# Detection of latent tuberculosis infection among migrant farmworkers along the US-Mexico border

**DOI:** 10.1186/s12879-016-1959-3

**Published:** 2016-11-03

**Authors:** E. Oren, M. H. Fiero, E. Barrett, B. Anderson, M. Nuῆez, F. Gonzalez-Salazar

**Affiliations:** 1 Department of Epidemiology & Biostatistics, Mel and Enid Zuckerman College of Public Health, University of Arizona, 1295 N. Martin Ave., P.O. Box 245211, Tucson, AZ 85724 USA; 2 Yuma County Health Services District, 2200 W 28th St, Yuma, AZ 85364 USA; 3 Mexican Social Security Institute, Juárez, México City Mexico; 4 University of Monterrey, Avenida Ignacio Morones Prieto 4500 Pte., Jesús M. Garza, 66238 San Pedro Garza García, NL Mexico

**Keywords:** Latent tuberculosis infection, Interferon-gamma release assay, Tuberculin skin test, Migrants

## Abstract

**Background:**

Migrant farmworkers are among the highest-risk populations for latent TB infection (LTBI) in the United States with numerous barriers to healthcare access and increased vulnerability to infectious diseases. LTBI is usually diagnosed on the border using the tuberculin skin test (TST). QuantiFERON-TB Gold In-Tube (QFT-GIT) also measures immune response against specific *Mycobacterium tuberculosis* antigens. The objective of this study is to assess the comparability of TST and QFT-GIT to detect LTBI among migrant farmworkers on the border, as well as to examine the effects of various demographic and clinical factors on test positivity.

**Methods:**

Participants were recruited using mobile clinics on the San Luis US-Mexico border and tested with QFT-GIT and TST. Demographic profiles and clinical histories were collected. Kappa coefficients assessed agreement between TST and QFT-GIT using various assay cutoffs. Logistic regression examined factors associated with positive TST or QFT-GIT results.

**Results:**

Of 109 participants, 59 of 108 (55 %) were either TST (24/71, 34 %) or QFT-GIT (52/106, 50 %) positive. Concordance between TST and QFT-GIT was fair (71 % agreement, ĸ = 0.38, 95 % CI: 0.15, 0.61). Factors associated with LTBI positivity included smoking (OR = 1.26, 95 % CI–1.01–1.58) and diabetes/high blood sugar (OR = 0.70, 95 % CI = 0.51–0.98).

**Discussion:**

Test concordance between the two tests was fair, with numerous discordant results observed. Greater proportion of positives detected using QFT-GIT may help avoid LTBI under-diagnosis. Assessment of LTBI status on the border provides evidence whether QFT-GIT should replace the TST in routine practice, as well as identifies risk factors for LTBI among migrant populations.

**Electronic supplementary material:**

The online version of this article (doi:10.1186/s12879-016-1959-3) contains supplementary material, which is available to authorized users.

## Background

Tuberculosis (TB) on the United States (US)-Mexico border is a disease of overcrowding, poverty, social exclusion, and lack of opportunity for human development [1, 2]. The US border region, defined by the La Paz Agreement as the area of land that stretches 100 km (62.5 miles) to the north and south of the international border with Mexico [3], is medically underserved, has higher uninsured rates, inequitable health conditions and some of the fastest-growing metropolitan areas [4]. The US-Mexico border states account for 30 % of total registered TB cases in both the US and Mexico [1, 5]. Yuma County, in which the San Luis border region is located, has a TB incidence rate 3–5 fold higher than the rest of the state of Arizona [6]. Border populations are at an increased risk of prolonged infectiousness, inadequate access to TB treatment, and multi-drug-resistant tuberculosis [7–9]. Infection with *Mycobacterium tuberculosis* bacilli can lead to latent tuberculosis (LTBI), in which the a patient is asymptomatic of disease [10]. Patients with LTBI are not contagious. If the host immune system is stressed or the patient develops an immunocompromised system (e.g. due to malnutrition, cancer, diabetes or HIV), LTBI can progress to active TB disease, where the patient is symptomatic and contagious [11, 12]. The overall lifetime risk of LTBI progressing to active TB infection is estimated to be 5–10 % but can be higher in high-risk populations such as those in the border region [11, 13–15]. Migrant farmworkers are among the highest-risk populations for LTBI in the US with an estimated six-fold higher risk of developing active TB compared to the average US worker [16]. Limited access to healthcare and health insurance, language barriers, poor education, long working hours, and political, social and economic disenfranchisement have been identified as barriers to healthcare and increased vulnerability to infectious diseases [17]. For example, our previous study along the border area states of Nuevo Leon and Tamaulipas, Mexico, identified rates of LTBI at 19 % (TB skin test) and 38 % (QFT-GIT), depending on diagnostic method [18]. However, while persons who have immigrated from TB-endemic regions of the world are recommended testing for LTBI, as is follow-up among immigrants with suspected TB, no policies exist for screening for LTBI among migrants along the border [19, 20].

There are two main diagnostic tools to detect LTBI: the tuberculin skin test (TST) and the interferon gamma release assays (IGRA) such as the Quantiferon TB gold-In-Tube (QFT-GIT). Both methods have strengths and limitations. The TST is an old tool that has cross-reactive or inadequate responses due to Bacillus Calmette-Guerin (BCG) vaccination (common in Mexico), environmental mycobacterium exposure (common in border soil samples), and compromised immune systems [15, 21–26]. Due to cross-reactivity, the TST can lead to false positive results which strain important public health resources; this may result in undue screening and treatment. The QFT-GIT is an IGRA test that measures the immune response to TB proteins in whole blood and is useful for individuals who have received the BCG vaccination in the past, as it is not cross-reactive like the TST (and hence more specific). As it requires a single participant visit for the blood draw, it is useful for those who may have a difficult time returning for the TST reading [27]. Few studies have evaluated the use of these tests in detecting LTBI in the US-Mexico border region, and none in Southwest Arizona [28]. Additionally, studies have identified the prevalence of LTBI in high-risk international border populations, but few have investigated the prevalence of LTBI in migrant populations who cross the US Mexico border for farmwork. Others have shown the prevalence of LTBI among migrant residents in the Baja California region to be close to 40 % [28]. Understanding the rates of infection in migrant farmworker populations will help guide future treatment and health needs of these groups [17], of particular importance given the more than three million estimates migrant and seasonal workers in the US [29].

Given this population’s large potential reservoir of LTBI, and the lack of available information regarding LTBI, the main objective of this work is to assess the utility and comparability of the two LTBI screening tests, TST and QFT-GIT, to detect LTBI among the migrant farmworker population in the Yuma/San Luis region in the state of Arizona in the United States. Second, we aim to examine the effects of various demographic (e.g. age) and clinical (e.g. smoking) factors on test positivity and TST/QFT-GIT discordance.

## Methods/design

### Setting and population

Migrant farmworkers (*N* = 109) were recruited and enrolled from March 2014 through November 2015 as they were crossing the San Luis, Arizona, border. Farmworkers were restricted to migrant workers, using the definition provided by the Migratory Health Network: “principal employment in agriculture on a seasonal basis, and employed as such within the last 24 months.” Individuals <18 years old, with current self-reported or diagnosed active tuberculosis disease or HIV, or known pregnancy were excluded. All participants were recruited at an outdoor park near the border crossing using a mobile clinic. The park is frequented by migrant farmworkers and passed through as one crosses the border. The study was advertised through the distribution of flyers to farmworkers at work in the field and in their living quarters. In addition, some participants were randomly approached and the study verbally explained while on their way home from work close to the recruitment site. All participants signed an informed consent with all study materials presented in Spanish. The study was approved by the University of Arizona’s Institutional Review Board. Monetary incentives in the amount $10 were given to all participants at the time of testing and an additional $5 provided at the time of TST read. Participants were free to withdraw from the study at any time.

### Questionnaire

Socio-demographic and clinical data were derived through individual interviews by trained public health staff using a standardized survey. The survey included demographic information (e.g. age, gender), TB exposure history, assessment of current infection and disease, and risk factors (housing conditions, crowding, co-morbidities such as diabetes, and behavioral risk factors such as smoking, excess alcoholic beverages (defined as five or more drinks in a sitting), or drug use.) Educational information was provided both verbally and in writing regarding LTBI in Spanish.

### Procedures

Participants were tested for LTBI by TST and QFT-GIT (for quantification of interferon gamma release). Trained nursing personnel from the Yuma Health Services District, who were blinded to the patient’s clinical details and TST result, performed the QFT-GIT test. Initially, the blood was placed into 3 different tubes containing 1 ml each; the first did not contain antigens (negative control), the second tube contained TB antigens (test) and the third contained phytohaemaglutinin (mitogen or positive control). Peripheral blood samples were then processed 6 to 8 h after sampling from the patient. The incubation time was 18–24 h at 37 °C. Interferon gamma production (IU/mL) was determined by ELISA. The results were considered positive, negative or indeterminate according to the criteria established in the manufacturer’s software (QFT Analysis Software v2.7, Qiagen). Once the blood was removed to perform the QFT-GIT test, the TST was performed using the Mantoux method, using 0.1 mL (2 tuberculin units) of purified protein derivative RT23 (Statens Serum Institute; Copenhagen, Denmark) in the middle of the anterior face of the forearm, with participants instructed to return to the same location 48–72 h for evaluation by experienced clinical staff. To read the TST, the transverse diameter of the induration was measured in mm. The TST reaction was scored as positive if the induration diameter was > 10 mm. Individuals were considered to have a diagnosis of LTBI if they were asymptomatic without clinical evidence of active tuberculosis, but had a positive QFT-GIT and/or TST positive reaction. All participants with a positive test were referred for further follow-up to staff at the Yuma Health Services District (either on-site or by phone call or letter), with provision of all services offered free of charge.

### Statistical analysis

Questionnaire data (as noted above) were collected and then entered into RedCAP, a secure data collection platform. The concordance between the QFT-GIT and TST tests was calculated using statistical kappa (κ). Kappa coefficients assessed agreement between TST and QFT-GIT. Strengths of agreement were considered ‘poor’, *κ* ≤ 0.20, ‘fair’, 0.20 < *κ* ≤ 0.40, ‘moderate’, 0.40 < *κ* ≤ 0.60, ‘good’, 0.60 < *κ* ≤ 0.80 and ‘very good’, 0.80 < κ ≤ 1.00. Sensitivity and specificity could not be calculated, as there is currently no gold standard for LTBI diagnosis. We computed Spearman's correlation coefficient to measure the association between the proportion of positive QFT-GIT with TST reactivity. Logistic regression was used to estimate unadjusted odds ratios (OR) to examine factors associated with positive TST and/or QFT-GIT results. As a sensitivity analysis, we increased the QFT-GIT assay (IU/mL) cutoff value from 0.35 to 1.0. This did not substantially change results (see Additional file 1 for full report of sensitivity analysis). A *p*-value < 0.05 was considered statistically significant. R version 3.2.3 was used for statistical analysis.

## Results

**Fig. 1:**
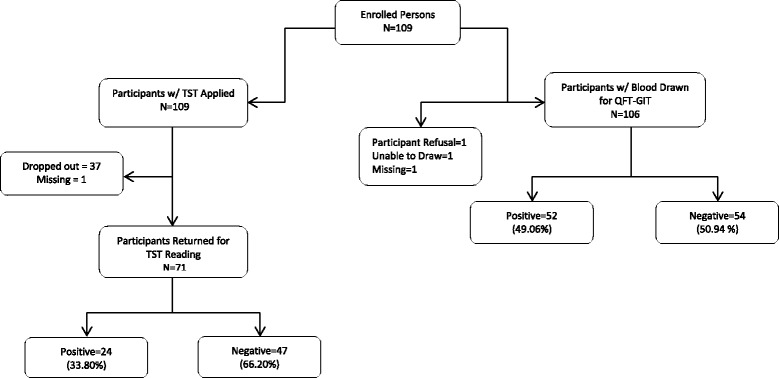
Participant Flow Diagram

Figure 1 shows the flow diagram of the participants who returned for TST reading and had blood drawn for QFT-GIT. A total of 109 participants enrolled in the study and had TST applied. Of these, 108 (99 %) completed procedures for at least one of the two tests, of whom 59 (54.6 %) had a positive test result by at least one of the tests. Of 71 participants who returned for TST reading, 24 (33.8 %) tested positive and 47 (66.2 %) tested negative. Among the 106 participants who had their blood drawn for QFT-GIT, 52 (49.1 %) tested positive and 54 (50.9 %) tested negative.

Most of the participants were male (76.9 %) at an average age of 46.8 years (Table 1). The majority of participants were working full time (61.2 %) and did not have health insurance (67.7 %). Fifty-three participants (60.9 %) reported excess alcohol use, 29 (33.3 %) reported being a current or former smoker, and eight (9.3 %) reported current or former injection drug use. Ten of 85 participants (13.3 %) with a response reported being diagnosed with diabetes/high blood sugar. We compared characteristics between participants who had TST read and those who did not, and did not find any significant differences (data not shown).

**Table 1:** Participant Characteristics (*N* = 109)

Characteristic	Number	Percent
Demographic Characteristics:		
Gender^a^		
Male	83	76.9
Female	25	23.1
Age: 46.8 ± 15.2 (mean ± SD)^a^		
18–34 years	31	28.7
35–49 years	25	23.1
50–64 years	38	35.2
65–75 years	14	13.0
Education^b^		
None or some primary	18	18.4
Completed primary	24	24.5
Some secondary	37	37.8
Completed high school or above	19	19.4
Employment (hours/week)^b^		
Full time (≥30)	60	61.2
Part time (<30)	20	20.4
Unemployed/Not working	18	18.4
Health insurance^c^		
Yes	30	30.3
No	67	67.7
Do not know	2	1.0
Years of farmwork: 19.2 ± 13.6 (mean ± SD)^d^		
< 5 years.	21	22.3
5–10 year.	15	16.0
10–25 years.	28	29.8
> 25 years.	30	31.9
No. total persons per household^b^		
1–3	50	51
≥ 4	48	49
No. total persons sleeping in same room^b^		
1	32	32.7
2	42	42.9
≥ 3	24	24.5
Risk Factors:		
Prior BCG vaccination^e^		
Yes	54	74.0
No	11	15.1
Do not know	8	11.0
Excess alcohol use^f^		
Yes	53	60.9
No	33	37.9
Refuse to answer	1	1.1
Current or former smoker^f^		
Yes	29	33.3
No	56	64.4
Do not know	1	1.1
Refuse to answer	1	1.1
Current or former injection drug use^g^		
Yes	8	9.3
No	78	90.7
Diabetes/High blood sugar^f^		
Yes	10	13.3
No	63	84.0
Do not know	2	2.7

*Abbreviations*: *SD* standard deviation, *BCG* Bacillus Calmette-Guerin

^a^One missing

^b^11 missing

^c^10 missing

^d^15 missing

^e^36 missing

^f^22 missing

^g^23 missing

Among 68 participants who returned for TST reading and had their blood drawn for QFT-GIT, 16 (23.5 %) tested positive on both tests and 32 (47.1 %) tested negative on both tests (Table 2). Of 44 participants who tested negative on TST (<10 mm), 12 (27 %) tested positive using QFT-GIT (one of three with an induration <10 mm testing positive). Overall agreement rate of results among participants who had TST and QFT-GIT results was fair 70.6 % (*k* = 0.38, 95 % CI = 0.15–0.61, *p* = 0.001). There was a trend towards an increased proportion of positive QFT-GIT with increasing TST reactivity (Spearman’s correlation coefficient 0.43, *P* < 0.001). When participants were limited to those with a known positive history of BCG vaccination, the results were unchanged.

**Table 2:** Comparison of TST and QFT-GIT results among the 68 participants who returned for TST reading and had their blood drawn for QFT-GIT

	QFT-GIT		
TST	Positive	Negative	Total
Positive	16	8	24
Negative	12	32	44
Total	28	40	68

Table 3 shows associations between participant characteristics and a positive result based on either test or both TST and QFT-GIT. Among 71 participants who returned for TST reading, current or former smokers were more likely to have positive results using TST compared to non-smokers (OR = 1.35, 95 % Confidence Interval (CI) = 1.05–1.74) (Table 3). Current or former injection drug use (OR = 1.59, 95 % CI = 1.03–2.46) was associated with detecting a positive TST result. Participants with diabetes/high blood sugar were less likely to have a positive TST result than participants who did not have diabetes/high blood sugar (OR = 0.69, 95 % CI = 0.49–0.97). No covariates were associated with a positive QFT-GIT result among those who had their blood drawn for QFT-GIT.

**Table 3:** Frequency, unadjusted odds ratios (OR) and 95 % confidence intervals (CI) for covariates associated with positive results by TST, QFT-GIT, either TST or QFT-GIT and both TST and QFT-GIT

	TST ≥ 10 mm	QFT-GIT positive	TST ≥ 10 mm or QFT-GIT positive	TST ≥ 10 mm and QFT-GIT positive
	(*N* = 71)	(*N* = 106)	(*N* = 108)	(*N* = 68)
Demographic Characteristics:				
Gender				
Male	Reference	Reference	Reference	Reference
Female	0.99(0.76–1.28)	0.97(0.77–1.22)	0.98(0.78–1.23)	1.00(0.78–1.26)
Age				
18–34 years	Reference	Reference	Reference	Reference
35–49 years	1.11(0.82–1.65)	0.77(0.59–1.00)	0.91(0.69–1.19)	0.92(0.69–1.22)
50–64 years	1.06(0.78–1.44)	1.04(0.82–1.31)	1.03(0.81–1.31)	1.05(0.80–1.39)
65–75 years	1.13(0.78–1.65)	1.10(0.80–1.52)	1.15(0.83–1.60)	1.10(0.78–1.57)
Education				
None or some primary	Reference	Reference	Reference	Reference
Completed primary	1.18(0.82–1.68)	1.02(0.74–1.41)	1.05(0.77–1.45)	1.14(0.82–1.69)
Some secondary	1.09(0.79–1.50)	1.02(0.76–1.37)	1.07(0.80–1.44)	1.02(0.75–1.38)
Completed high school or above	1.12(0.75–1.67)	1.12(0.80–1.58)	1.12(0.80–1.58)	1.13(0.77–1.63)
Employment				
Full time (≥30)	Reference	Reference	Reference	Reference
Part time (<30)	1.19(0.90–1.58)	0.98(0.76–1.26)	1.11(0.86–1.44)	1.00(0.78–1.29)
Unemployed/Not working	1.07(0.80–1.43)	1.19(0.90–1.58)	1.11(0.84–1.48)	1.30(0.98–1.72)
Health insurance				
Yes	Reference	Reference	Reference	Reference
No	1.12(0.87–1.45)	1.06(0.85–1.32)	1.14(0.91–1.42)	1.03(0.81–1.30)
Years of farmwork				
< 10 year.	Reference	Reference	Reference	Reference
10–25 years.	1.02(0.77–1.35)	0.87(0.68–1.12)	0.89(0.69–1.14)	0.99(0.76–1.29)
> 25 years.	1.11(0.84–1.46)	1.12(0.88–1.44)	1.13(0.89–1.45)	1.09(0.84–1.42)
No. total persons per household				
1–3	Reference	Reference	Reference	Reference
≥ 4	0.88(0.70–1.10)	1.06(0.87–1.30)	1.02(0.83–1.25)	0.92(0.75–1.13)
No. person sleep in same room				
1	Reference	Reference	Reference	Reference
2	0.88(0.68–1.08)	0.83(0.65–1.04)	0.89(0.70–1.13)	0.79(0.63–1.00)
≥ 3	0.79(0.57–1.08)	0.99(0.75–1.29)	0.92(0.70–1.22)	0.86(0.64–1.15)
Risk Factors:				
Excess alcohol use				
No	Reference	Reference	Reference	Reference
Yes	1.06(0.83–1.35)	0.88(0.71–1.10)	0.88(0.71–1.10)	1.02(0.82–1.27)
Current or former smoker				
No	Reference	Reference	Reference	Reference
Yes	1.35(1.05–1.74)*	1.06(0.84–1.33)	1.26(1.01–1.58)*	1.02(0.80–1.30)
Current or former injection drug use				
No	Reference	Reference	Reference	Reference
Yes	1.59(1.03–2.46)*	1.42(0.99–2.03)	1.28(0.89–1.84)	1.82(1.26–2.64)*
Diabetes/High blood sugar				
No	Reference	Reference	Reference	Reference
Yes	0.69(0.49–0.97)*	0.78(0.56–1.08)	0.70(0.51–0.98)*	0.78(0.57–1.06)

*P-value < 0.05

Among 108 participants who returned for TST reading or had blood drawn for QFT-GIT, current or former smoking was associated with an increased likelihood of a positive result (OR = 1.26, 95 % CI = 1.01–1.58). Participants with diabetes/high blood sugar were 30 % less likely to have either a positive TST or QFT-GIT result than participants who did not have diabetes/high blood sugar (OR = 0.70, 95 % CI = 0.51–0.98). Among 68 participants who returned for TST reading and had their blood drawn for QFT-GIT, current or former injection drug users were more likely to have positive results from both TST and QFT-GIT compared to participants who were not injection drug users (OR = 1.82, 95 % CI = 1.26–2.64) (Table 3).

We also looked at predictors of discordance between TST and QFT-GIT results (positive TST and negative QFT-GIT/negative TST and positive QFT-GIT). No covariates were found to be associated with obtaining discordant results between the two tests among participants who completed both.

## Discussion

In this study, the TST and QFT-GIT assays were evaluated in the diagnosis of LTBI in a sample of migrant farmworkers working on the US side of the US-Mexico border. A higher proportion of individuals tested positive by the QFT-GIT, with many of these individuals displaying a negative TST. Test concordance between the two tests was fair.

If we consider a positive result with either test to indicate LTBI, our findings of approximately half of the sample testing positive (55 %) is much higher than that previously published in the general population in the US (5 %) as well as a relatively high estimate, when compared with existing border and migrant estimates on the US and Mexico border regions of between 30–40 % [18, 28, 30, 31]. Low socioeconomic status in other US populations has previously been linked to increased rates of TB disease transmission (and while unmeasured, new infections) [26]. We may have also encountered a population with higher prior exposure to TB, a greater proportion of previously BCG-vaccinated individuals, or differing immune competency and reactivity. The latter factors may also explain the lower prevalence (24 %) of individuals testing positive concurrently on both tests.

The QFT-GIT test results unexpectedly demonstrated a higher prevalence of LTBI compared with TST. While these findings could be considered surprising in a highly BCG-vaccinated population, they are consistent with findings in other high-risk groups, both in our border work in Mexico [18] and elsewhere [32, 33]. Others have noted that IGRAs are sensitive for detecting LTBI in various immuno-compromised populations [34, 35]. Of those participants who tested negative on TST approximately a quarter tested positive using QFT-GIT. Individuals unreactive to TST due to diminished immune response may thus benefit from the QFT-GIT or other IGRA [34]. Anergy to TST may also have occurred due to malnutrition, absence of infection or technical problems with the test. It is possible that QFT-GIT results may be falsely positive if the 0.35 IU/ml cut-off is too low. However, in our sensitivity analyses with a 1.0 IU/ml cut-off, the proportion positive did not shift much. Given that other high-risk populations, such as health care workers, can progress to active TB with QFT responses as low as 0.35–0.7 IU/ml, we elected to report primary study findings using the lower cut-off [36].

As per the authors’ prior results from the Mexico side of the border, we found the TST and QFT-GIT to show a low level of overall concordance [18, 31]. Eight participants who had positive TST results (33 %) were negative on QFT-GIT. This is similar to both empirical and meta-analytic findingsfrom around the world, where a high proportion of individuals with large skin test indurations tested negative on QFT or a comparably low kappa statistic was noted [32, 37–39]. We expect that these findings represent false positive TST results, due to either BCG or non-tuberculous mycobacteria (NTM) cross-reactivity. Positive QFT results may also represent cross-reactivity with NTM, though we were unable to test this hypothesis [40].

In our study, current or former smokers, as well as injection drug users, were more likely to have positive TST results compared to non-users. Participants with diabetes/high blood sugar were less likely to have a positive TST result than participants who did not have diabetes/high blood sugar. Similarly, current or former smoking was associated with an increased likelihood of getting a positive result from either TST or QFT-GIT when receiving both tests. Participants with self-reported diabetes/high blood sugar were less likely to have either a positive TST or QFT-GIT result, as reported in other studies [41].

While diabetes is associated with a three-fold risk of progression a risk factor for development of active TB [23], the mechanistic link between diabetes and LTBI has not been closely examined. Diabetes suppresses the immune system, with decreased levels observed of *M. tuberculosis*-specific antigen-stimulated IFN-γ production in whole blood of diabetes patients with LTBI, potentially resulting in an anergic response, as we hypothesize occurred in our study [42, 43]. Current smoking has been previously associated with LTBI, with the thought that macrophages from smokers less competent in controlling intracellular *M. tuberculosis* in comparison to never smokers [44]. Likewise, drug use has been associated with a higher prevalence of LTBI, likely due to a combination of physiological effects of drug use, as well as environmental and risky behaviors [45].

Limitations of our study include the cross-sectional nature of the results, as well as the ability to generalize these local results to other parts of the border, where there are diverse farmworker groups and socioeconomic conditions. We may have recruited a biased sample of participants differing from the broader farmworker population with regards to factors such as education, fear and trust. We also recognize that for some risk factors, levels of non-response were high and precluded the ability to establish associations with high precision or to run adjusted analyses. For example, missing BCG vaccination status for 40 % of the sample precluded stratification of the results by BCG status. Missing TST reads on a large proportion of the sample prevented us from being able to compare the broader group by TST/QFT status. Finally, data were self-reported and morbidity measures such as diabetes were not objectively verified. Further studies could examine the two screening tests in a larger, more representative sample, as well as evaluate host factor associations with test response.

## Conclusions

Our work provides comparative data between the QFT-GIT and TST on the border, including utility of the two tests and recommendations among the populations studied. A higher proportion of individuals tested positive by the QFT-GIT, with fair concordance between the two tests. While we were unable to determine whether QFT-GIT was a more specific test, the importance of a test that does not cross-react with BCG vaccination is notable in this population, with concerns regarding both costs and adverse effects of LTBI treatment that might occur among false positives. As per US recommendations, targeted testing activities should be conducted among groups at high risk for progression to active TB disease (such as priority groups that are medically underserved or immune-compromised), with intent to treat if LTBI is detected [46]. Given the observed risk factors for progression to active TB disease, it is important to provide populations on the border with information around TB and LTBI, including trainings and informational materials. Additional research on LTBI screening among different high-risk subpopulations on the border is also needed.

## Additional file

Additional file 1: Comparison of TST and QFT-GIT results among the 68 participants who returned for TST reading and had their blood drawn for QFT-GIT (QFT-GIT assay 1.0 IU cut-off). (PDF 294 kb)

## Abbreviations

BCG: Bacillus Calmette-Guerin
IGRA: Interferon gamma release assays
LTBI: Latent Tuberculosis Infection
NTM: Non-tuberculous mycobacteria
PIMSA: Programa Internacional de Migracion en Salud
QFT-GIT: QuantiFERON-TB Gold In-Tube
TB: Tuberculosis
TST: Tuberculin skin test
US: United States
