# Supplementary material for: Detection of latent tuberculosis infection among migrant farmworkers along the US-Mexico border
Source: BMC Infect Dis. 2016 Nov 3;16:630. doi: 10.1186/s12879-016-1959-3 (PMC5096297; doi:10.1186/s12879-016-1959-3)
Supplement: Additional file 1: — Comparison of TST and QFT-GIT results among the 68 participants who returned for TST reading and had their blood drawn for QFT-GIT (QFT-GIT assay 1.0 IU cut-off). (PDF 294 kb) [file 12879_2016_1959_MOESM1_ESM.pdf]

Supplementary Appendix

Sensitivity analysis

We carried out a sensitivity analysis to examine results after increasing the QFT-GIT assay (IU/mL) from 0.35 to 1.0. The number of participants who had a positive QFT-GIT result decreased from 51 (46.8%) to 30 (27.5%).

Table 1 shows a comparison of the TST and QFT-GIT results. Among the 68 participants who returned for TST reading and had their blood drawn for QFT-GIT, 13 (19.12%) tested positive on both tests and 37 (54.41%) tested negative on both tests. The overall agreement rate of results among participants who had TST applied and had their blood drawn for QFT-GIT increased from 70.6% to 73.5% (k = 0.40, 95% Confidence Interval (CI) = 0.16-0.64, p = 0.002) (Table 1).

Results of the logistic regression analyses evaluating associations between covariates and detecting a positive result remained similar after adjusting the QFT-GIT assay. Among participants who had their blood drawn for QFT-GIT, injection drug use (Odds Ratio (OR) = 1.45, 95% Confidence Interval (CI) = 1.05-2.01) was associated with an increased likelihood of detecting a positive QFT-GIT result. Among participants who returned for TST reading or had their blood drawn for QFT-GIT, those with diabetes/high blood sugar were more likely to obtain a positive TST or QFT-GIT result (OR = 0.71, 95% CI = 0.52-0.99) compared to those who did not have diabetes/high blood sugar. Participants who reported current or former injection drug use were more likely to have a positive TST or QFT-GIT result compared to those who did not report injection drug use (OR = 1.45, 95% CI = 1.02-2.07). Among those who had returned for TST reading and had their blood drawn for QFT-GIT, current or former injectioon drug use (OR = 1.83, 95% CI = 1.26-2.65) was associated with an increased likelihood of detecting both a positive TST and positive QFT-GIT result.

Table 1. Comparison of TST and QFT-GIT results among the 68 participants who returned for TST reading and had their blood drawn for QFT-GIT, after increasing the QFT-GIT assay (IU/mL) from 0.35 to 1.0.

TST	QFT-GIT		
	Positive	Negative	Total
Positive	13	11	24
Negative	7	37	44
Total	20	48	68
